# Pattern of opioid prescriptions among patients with breast, lung, and colorectal cancer diagnosed with pre-existing chronic non-cancer pain

**DOI:** 10.1371/journal.pone.0352907

**Published:** 2026-07-29

**Authors:** Safalta Khadka, J Douglas Thornton, Khalid M Kamal, Gerald Higa, Kimberly M. Kelly, Sabina O. Nduaguba

**Affiliations:** 1 Department of Pharmaceutical Systems and Policy, School of Pharmacy, West Virginia University, Morgantown, West Virginia, United States of America; 2 Prescription Drug Misuse Education and Research (PREMIER) Center, College of Pharmacy, University of Houston, Houston, Texas, United States of America; 3 Department of Clinical Pharmacy, West Virginia University, School of Pharmacy, Morgantown, West Virginia, United States of America; 4 Department of Preventive Medicine, The University of Tennessee Health Science Center, Memphis, Tennessee, United States of America; 5 Mary Babb Randolph Cancer Center, West Virginia University, Morgantown, West Virginia, United States of America; Auburn University, UNITED STATES OF AMERICA

## Abstract

**Background:**

Chronic non-cancer pain (CNCP) due to comorbid conditions presents a barrier to optimal pain management in patients with cancer. While opioid is widely used for the treatment and management of CNCP, it is also associated with misuse and addictions. Therefore, we determined the impact of CNCP on opioid prescribing patterns in patients with the three most common cancer types- breast, lung, and colorectal cancers.

**Methods:**

A retrospective cohort study was conducted utilizing Surveillance Epidemiology and End Results (SEER)-Medicare (2006–2019) linked database. Patients, at least 66 years old with histologically confirmed incidental diagnoses of breast, lung, or colorectal cancer from 2007 to 2017, were included in the study. Patients with cancer in baseline period, in palliative care, or those who died in the follow-up period were excluded from the study. Patients with CNCP were identified in the baseline period. Patients without CNCP during the study period were included in the no-CNCP group. The follow-up period was one year after cancer diagnosis (index date) and the primary outcome was opioid prescribing patterns defined as: any opioid use, high-dose opioid use, total count of opioid prescriptions filled, and chronic opioid use. Inverse probability weighting (IPTW) method was used to balance the covariates (age at the cancer diagnosis time, sex, race, ethnicity, marital status, cancer type, cancer stage, metropolitan status, year of cancer diagnosis, chronic cancer-treatment-related pain, neoplasm pain, and cancer treatment modalities) between the CNCP and no-CNCP group. Logistic and Poisson regressions were used to compare opioid use and opioid prescription patterns among the two groups.

**Results:**

A majority of patients included in the study were breast cancer (CNCP = 38230, no-CNCP = 56157), lung cancer (CNCP = 17255, no-CNCP = 24602), colorectal cancer (CNCP = 16660, no-CNCP = 29037). Among cancer patients, the prevalence of CNCP was highest in those with lung cancer (41.2%) followed by breast cancer (40.1%) and colorectal cancer (36.4%). Descriptive analysis demonstrated patients with lung cancer had consistently higher rates across opioid prescribing patterns, followed by patients with colorectal and breast cancer. The result of logistic regression after weighting showed that the odds of any opioid use were higher among lung cancer patients with CNCP (OR 1.5; 95% CI 1.5–1.6; p < 0.01) compared to the no-CNCP group with lung cancer. Patients with breast cancer and CNCP had higher odds of chronic opioid use followed by lung cancer and colorectal cancer (OR 2.7; 95% CI 2.6–2.8; p < 0.01, OR 2.4, 95% CI 2.3–2.5; p < 0.01, and OR 2.4; 95% CI 2.3–2.5; p < 0.01) respectively compared to patients without CNCP but respective cancer.

**Conclusion:**

CNCP adds an additional burden in opioid prescription in active cancer patients which necessitates a meticulous approach to opioids prescribing.

## Introduction

Globally, chronic pain affects more than 30% of people, and in the United States, prevalence of chronic pain was 24.3% in 2023 [1]. Although definitions vary widely, chronic pain is defined as a condition in which a person has pain most days or every day for longer than three months [2–4]. Additionally, among patients with chronic pain, 34.9% of adults had high-impact chronic pain (chronic pain that significantly impacts the patient’s quality of life and frequently limits daily life activities) [1,5]. The prevalence rates of chronic pain differ and are higher in women, people living in rural areas, and people with lower socioeconomic status [1]. Similarly, elderly patients are particularly vulnerable to chronic pain due to physiologic changes, higher comorbidity burden, and increased risk of polypharmacy. Furthermore, the presence of chronic pain can severely impact multiple aspects of health such as quality of life of patients, sleep and mental health, cardiovascular health, and brain function [6–9]. Chronic pain also imposes substantial economic burden on the patients and the health care system [10]. The annual direct medical cost and productivity loss among patients with chronic pain was estimated to be $530.6 billion and $192.2 billion in 2021, and the annual cost of prescription opioids was estimated to be $3.57 billion in 2010 [11, 12]. Although more recent national estimates of the broader opioid crisis are available [13,14], updated analysis on economic impact isolating the direct medical cost of medically prescribed opioids remain limited.

Pain is highly prevalent among patients with cancer and can stem from the neoplasm itself and associated cancer treatment. The overall prevalence is 44.5% but varies depending on the cancer stage and cancer type, with prevalence up to 54.6% in advanced, metastatic and terminal cancer patients [15]. For patients receiving cancer treatment, 55% report pain, and the prevalence of pain is still around 39% even after curative treatment [16]. Breast, lung and colorectal cancer are among commonly prevalent cancer in US with variations in disease trajectories, survival patterns, and treatment strategies [17–19]. Despite these differences, chronic pain remains common [20–22]. Although prostate cancer is also commonly prevalent among older adults, it was excluded due to distinct clinical trajectory and the high prevalence of bone metastasis [23], which may complicate interpretation of opioid use for comorbid chronic non-cancer pain (CNCP).

Elderly patients with cancer are at risk of increased CNCP [24,25] which may be attributable to conditions such as osteoporosis, migraine, neuropathic pain, arthritis, chronic back pain, and fibromyalgia causing chronic pain. The presence of CNCP associated with comorbid conditions presents challenges such as diagnostic complexity, risk of inappropriate prescribing, and polypharmacy in treatment and management of pain in cancer patients [26,27].

Opioids are the cornerstone for the treatment and management of pain among patients with cancer [26]. While opioids can have high clinical utility in certain scenarios, such as managing acute postoperative pain and pain in palliative care, they are also associated with serious adverse effects such as respiratory depression, opioid addiction, and opioid overdose deaths, particularly when overprescribed, misuse, or used inappropriately [28–30]. Opioid-related deaths increased from 0.52 to 0.66 per 100,000 between 2006 and 2016 among patients with cancer [31]. In the wake of the opioid epidemic in US, 106,699 drug overdose deaths were recorded in 2021 [32]. Among patients with cancer, the prevalence of opioid misuse is 12.3% [33] and non-cancer-related opioid use among patients with cancer is expected to increase [30,34]. Further, with the advancement in treatment and technology, the number of cancer survivors are increasing, who may experience either cancer-related or non-cancer related pain [35,36]. There is, therefore, a growing need for health care professionals to optimize pain management for patients with cancer in order to minimize misuse and adverse effects and ultimately improve health outcomes in this population.

Numerous guidelines and policies have been introduced to manage pain and opioid prescribing in the general population with chronic pain as well as in cancer patients [26,30,37–42]. However, a gap still exists for the patients with dual diagnosis, i.e., those with cancer and CNCP. For instance, the Centers of Disease Control and Prevention (CDC) guideline (2022) for prescribing opioids for the management of chronic pain excludes patients undergoing active cancer treatment [38]. Similarly, the National Comprehensive Cancer Network (NCCN) and American Society of Clinical Oncology (ASCO) guidelines, prepared specifically for managing cancer pain, has focused on cancer pain, cancer-treatment-related pain or overall pain. However, these guidelines do not extensively address the management of comorbid chronic pain conditions [26,42]. Additionally, most of the literature has studied opioid use for the management of chronic pain in cancer survivors but excluded patients with active cancer [43,44]. This is largely because opioids use in patients in active cancer are clinically complex and often guided by a primary emphasis on survival and symptom control. Additionally, few studies have examined active opioid use among patients with active cancer, but did not include elderly cancer patients [45–47]. Therefore, there is a substantial gap in evidence related to opioid prescribing in elderly patients with cancer and CNCP. This paucity of evidence on non-cancer chronic pain among elder patients with cancer creates a challenge for clinicians as they lack a clear direction on ways to integrate pain management in patients with dual diagnosis of cancer and chronic pain.

An understanding of the practice and prescription patterns of opioids in patients with CNCP and cancer is required to inform policy makers and practitioners who can, in turn, advocate for those with CNCP. In this study, we sought to assess the impact of CNCP on opioid prescribing patterns including opioid prescription rate, high dose of opioid prescriptions, and chronic opioid use in elderly patients with three most common cancer types- breast, lung, and colorectal cancers.

## Methods

### Data source

The study used the Surveillance Epidemiology and End Results-Medicare (SEER-Medicare) Linked Database from 2006–2019. SEER is a database created by the National Cancer Institute that collects information on patient demographics, tumor morphology, primary tumor site, tumor morphology, stage at diagnosis, and treatment [48]. The data covers 48% of the US population and is the only comprehensive source of information for cancer diagnosis and patient survival.

The linkage of SEER-Medicare data includes two large population-based sources of data and provides rich information on elderly patients (65 years and older) diagnosed with cancer and enrolled in Medicare. Medicare enrollment data includes individual patient level information, monthly indicators for enrollment in: Part A (inpatient), part B (outpatient), part C (Medicare Advantage – claims not available) and part D (prescription drugs). Medicare part A and part B claims data provide information on health services utilization such as outpatient services, inpatient services, hospice and home health care, skilled nursing facility care, physician/supplier services, infusion/injectable drugs, date of services used and cost of the services for elderly beneficiaries with FFS plans. The Medicare part D file includes data on payments and date of services for prescription drugs [48–50]. The data was accessed on April 20^th^, 2023 and the authors did not have access to information that could identify individual participants during or after data collection.

### Study design and population

This was a retrospective observational study and was reviewed and approved by the institutional review board (IRB) of West Virginia University before the study began.

**Fig 1:**
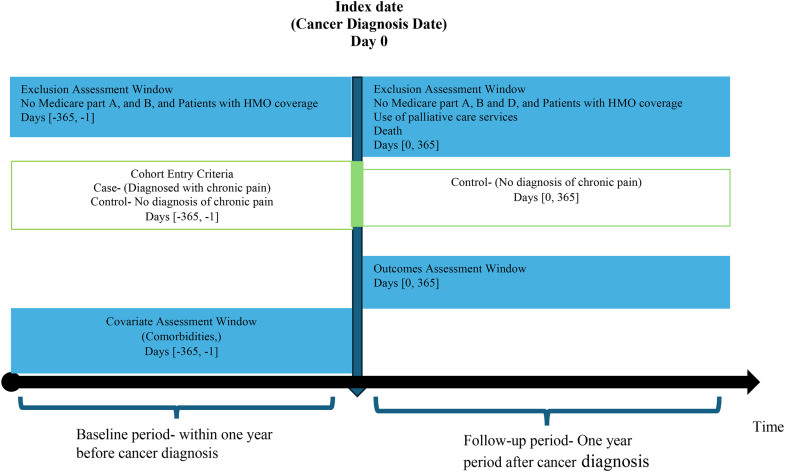
Study design schema.

The study included patients with the histologically confirmed and incident diagnosis of breast, lung, or colorectal cancer from Jan 2007 to Dec 2017 (Fig 1). The type of cancer was identified using International Classification of Disease for Oncology (ICD-O3) codes. Patients aged at least 66 years old at the time of diagnosis of cancer were included in the study. Furthermore, patients diagnosed with CNCP in the baseline period (one year before cancer diagnosis) were included using ICD-09 and ICD-10 codes, and the date of cancer diagnosis was the index date for the study. Patients were followed for one year after the index date. Patients without a CNCP diagnosis during the baseline period and follow-up study were considered the no-CNCP group. Furthermore, patients with cancer in baseline period, patients in palliative care (ICD-9 code- V66.7 and ICD-10 code-Z51.5), or those who died in the follow-up period were excluded from the study [51–53]. To further validate the codes for palliative care, we determined the survival days of patients in palliative care, and approximately 83% of patients had survival days less than six months. Additionally, any patients with Health Maintenance Organization (HMO) coverage and without continuous enrolment in Medicare Part A and Part B in the baseline period and Medicare part A, part B and part D in follow-up period were excluded from the study.

### Identification of CNCP patients

Patients with CNCP were identified using ICD-9 and ICD-10 codes. Tain *et al.*, have previously validated ICD-9 codes for medical conditions likely to be associated with chronic pain [54]. Based on the validated ICD-9 codes, we derived ICD-10 codes for CNCP using a forward and backward mapping method [55]. Further, the validated algorithm had two different categories – ‘highly likely category to represent chronic pain’ and ‘likely to represent chronic pain.’ Chronic pain was defined as a single occurrence of an ICD-9 and ICD-10 codes in the former category during the baseline period, and two or more occurrences of ICD-9 and ICD-10 codes in the latter category, separated by at least 30 days in the study period [54]. Patients meeting any of these criteria were included in the study. Patients without any single occurrences of ICD-9 and ICD-10 codes in both highly likely category and likely to represent the category during the baseline and follow-up period were included in the no-CNCP cohort.

### Patterns of opioid prescriptions

The patterns of opioid prescriptions were explored in terms of any opioid use, chronic opioid use, high dose of opioid prescriptions and total count of prescriptions. Any opioid use was defined as at least one claim for the opioid prescriptions during the follow up period. For chronic opioid use, previous Medicare-based studies used cumulative days’ supply threshold and varying permissible gap allowances [56–59]. We defined chronic opioid use as continuous opioid use for ≥90 days after cancer diagnosis date with a five-day permissible gap in claims for opioid prescriptions to capture sustained exposure [43,60]. Furthermore, an average morphine milligram equivalent (MME) per day was calculated to determine the extent of high dose of opioid use. Average MME was calculated from opioid dose using a CDC Conversion factor categorized in three categories: ‘<50 MME/day’, ’50–90 MME/day’, and ‘>90 MME/day’ [61,62]. High dose of opioid prescriptions was defined when the patients had prescription of average of >90 MME/day [43]. Further, the total count of opioid prescriptions filled were calculated among patients prescribed with opioids. Opioids were identified using the generic name from the Medicare part-D prescription drug event data files (S1 Table) and were selected based on the availability of MME conversion factors. We assumed that patients used opioids if they had claims for opioid prescriptions.

### Covariates

Socio-demographic variables such as age, sex, race, ethnicity, marital status, metropolitan status, year of cancer diagnosis and clinical variables such as type of cancer, cancer stage, cancer treatment modalities, and Charlson Comorbidity Index (CCI) were included as covariates. The variables were selected based on prior literature that showed association of these factors with opioid use [43,44,63–66]. Age, sex, race, ethnicity, and metropolitan status [67] were recorded at the time of diagnosis of cancer. CCI was obtained from the comorbidities recorded during the 12 months prior to index date (Fig 1) [68,69]. ICD-9 and ICD-10 were used to identify comorbid conditions to calculate CCI. Cancer treatment modality was defined as the first course therapy that occurred after cancer diagnosis. Cancer treatment modalities included four categories: ‘chemotherapy’, ‘radiotherapy’, ‘surgery’ and ‘more than one treatment’ and was derived from the SEER dataset. More than one treatment represents when patients receive combination of treatment modalities such as surgery and adjuvant chemotherapy, or combination of radiotherapy, surgery, and adjuvant chemotherapy during the first course therapy. Further, cancer pain was defined in two ways: chronic cancer-treatment-related pain (chronic pain associated with cancer treatment) and neoplasm pain (pain associated with tumor) after cancer diagnosis. Chronic cancer-treatment-related pain and neoplasm pain after cancer diagnosis were identified using ICD-9 and ICD-10 codes (S1 Table). Chronic cancer-treatment-related pain was identified using the two or more occurrences of ICD-9 and ICD-10 codes greater than or equal to 90 days period and was derived as binary variables based on the presence/absence of the condition [70–72]. Additionally, cancer stage was determined using SEER Summary 2000 [73] and was categorized into one of four categories: in situ, localized, regional, and distant.

### Analysis

Patients were followed for a one-year post-index date. In this retrospective observational study, patients were not randomly selected from CNCP and no-CNCP cohort, which could lead to potential selection bias in the study. To reduce the potential selection bias, propensity score (PS) analysis is a commonly used method. Inverse probability of treatment weighting (IPTW) is one way of application of PS, as it produces pseudo-population of comparable characteristics [74]. In this approach, PS were estimated to calculate probability weight ensuring balance in baseline characteristics between CNCP and no-CNCP groups. IPTW was conducted in two steps [75]. First, a PS was calculated for being in CNCP group using a multivariable logistic regression model [74]. The regression model included age at the cancer diagnosis time, sex, race, ethnicity, marital status, cancer type, cancer stage, metropolitan status, year of cancer diagnosis, chronic cancer-treatment-related pain, neoplasm pain, and cancer treatment modalities. In the next step, stabilized weights were calculated for each patient for both CNCP and no-CNCP group. Standard differences were compared before and after weighing to confirm the quality of the weighing process [76]. The standardized score of <10% in the matched dataset was considered a balance in covariates.

Baseline demographics were compared between CNCP and no-CNCP group before and after weighting group using chi-square test for the categorical variables and student T-test for continuous variables for each type of cancer. To explore the opioid prescription patterns, we determined the association of each of these outcomes 1) any opioid use, 2) high dose of opioid use, 3) chronic opioid use and 4) total opioid prescription count per patient, independently with the CNCP diagnosis. Chi-square and T-tests were used to determine the difference in opioid prescription patterns between CNCP and no-CNCP cohort. To further determine the difference in different cancer types within CNCP cohort, chi-square and ANOVA tests were used. The same analysis was applied within no-CNCP cohort as well. We used logistic regression models to determine the odds of opioids prescription between CNCP and no-CNCP group. Separate models were created for each outcome: any opioid use, high dose of opioid use, and chronic opioid use. Further, Poisson regression was used to analyze the total count of opioid prescription filled per patient. Results were presented in odds ratio and 95% Confidence Intervals (CI) for any opioid use, chronic opioid use, high dose of opioid use, and results for total count of opioid prescription filled per patient were presented in estimate with 95% CI. SAS 9.4 version was used for analysis.

## Results

### Sample characteristics before IPTW

Table 1 describes the sociodemographic characteristics and clinical characteristics of the study cohort. In total, 181,941 patients were included in the study, and 72,145 (39.6%) had CNCP. Patients in the study included breast cancer (CNCP = 38230 (40.5%), no-CNCP = 56157 (59.4%)), lung cancer (CNCP = 17255 (41.2%), no-CNCP = 24602 (58.7%)), colorectal cancer (CNCP = 16660 (36.4%), no-CNCP = 29037 (63.5%)). Most patients in the overall sample were in the age group 66–70 years in breast cancer and lung cancer cohort (n = 28,849, 30.5%, n = 12663, 30.2%). Among patients with colorectal cancer, 25.0% patients were in ≥83 years (n = 11,419). Furthermore, most of the patients were female (99.1% in breast cancer cohort, 56.49% in lung cancer cohort, and 56.9% in colorectal cancer cohort). The most common cancer treatment modalities at the start of therapy were more than one treatment (48.1%) in the breast cancer cohort, whereas the common treatment modalities in patients with lung cancer and colorectal cancer was surgery only (28.6% and 48.21%, respectively). Similarly, most patients were diagnosed at the localized cancer stage for breast (59.6%), lung (42.1%), and colorectal cancer cohorts (45.6%). Table 1 shows the characteristics of patients in CNCP and no-CNCP group among patients with breast, lung, and colorectal cancer.

**Table 1:** Characteristics of cohort.

Sociodemo graphic Characteristics	Breast cancer			Lung cancer			Colorectal cancer		
	No CNCP diagnosis (N = 56157)	CNCP diagnosis (N = 38230)	p-value	No CNCP diagnosis (N = 24602)	CNCP diagnosis (N = 17255)	p-value	No CNCP diagnosis (N = 29037)	CNCP diagnosis (N = 16660)	p-value
Age (n, column %)			<0.001			<0.001			<0.001
66-70 age	18051 (32.14)	10798 (28.24)		7675 (31.20)	4988 (28.91)		6898(23.76)	3328(19.98)	
71-74 age	12473 (22.21)	8083 (21.14)		6121 (24.88)	3927 (22.76)		5589(19.25)	2880(17.29)	
75-78 age	9711 (17.29)	6892 (18.03)		4829 (19.63)	3477 (20.15)		5209(17.94)	2951(17.71)	
79-82 age	7222 (12.86)	5400 (14.13)		3345 (13.60)	2583 (14.97)		4552(15.68)	2871(17.23)	
83 and above age	8700 (15.49)	7057 (18.46)		2632 (10.70)	2280 (13.21)		6789(23.38)	4630(27.79)	
Sex (n, %)			<0.001			<0.001			<0.001
Male	536 (0.95)	270 (0.71)		11483 (46.68)	6728 (38.99)		13271 (45.70)	6402 (38.43)	
Female	55621 (99.05)	37960 (99.29)		13119 (53.32)	10527 (61.01)		15766 (54.30)	10258 (61.57)	
Race (n, %)			<0.001			<0.001			<0.001
White	49123 (87.47)	33704 (88.16)		21568 (87.67)	15263 (88.46)		24641 (84.86)	14414 (86.52)	
Black	4024 (7.17)	2925 (7.65)		1555 (6.32)	1119 (6.49)		2241 (7.72)	1248 (7.49)	
Others	2719 (4.84)	1377 (3.60)		5571 (5.08)	821 (4.76)		1976 (6.81)	916 (5.50)	
Ethnicity (n, %)			0.84			0.94			0.0024
Non-Hispanic	52836 (94.09)	35981 (94.12)		23439 (95.27)	16442 (95.29)		26929 (92.74)	15576 (93.49)	
Hispanic	3321 (5.91)	2249 (5.88)		1163 (4.73)	813 (4.71)		2108 (7.26)	1084 (6.51)	
Marital Status (n, %)			<0.001			<0.001			<0.001
Married	18380 (32.73)	11509 (30.10)		9053 (36.80)	5770 (33.44)		10393 (35.79)	5301 (31.82)	
Wid/Sep/Div	16876 (30.05)	12064 (31.56)		6058 (24.62)	4639 (26.88)		7608 (26.20)	4682 (28.10)	
Never Married	3879 (6.91)	2655 (6.94)		1620 (6.58)	1095 (6.35)		2245 (7.73)	1193 (7.16)	
Metropolitian Status (n, %)			<0.001			0.95			<0.001
Metro	47412 (84.43)	32761 (85.69)		20646 (83.92)	14624 (84.75)		23915 (82.36)	14006 (84.07)	
Non-Metro	7764 (13.83)	4853 (12.69)		3523 (14.32)	2314 (13.41)		4514 (15.55)	2332 (14.00)	
Rural	971 (1.73)	609 (1.59)		429 (1.74)	315 (1.83)		606 (2.09)	320 (1.92)	
Types of Treatment (n, %)			<0.001			0.99			
No treatment	1276 (2.27)	966 (2.53)		1732 (7.04)	1298 (7.52)		1071 (3.69)	646 (3.88)	<0.001
Chemotherapy only	19 (0.03)	13 (0.03)		1370 (5.57)	868 (5.03)		416 (1.43)	201 (1.21)	
More than one	28657 (51.03)	18624 (48.72)		6048 (24.58)	3718 (21.55)		6092 (20.98)	2750 (16.51)	
Radiotherapy only	44 (0.08)	19 (0.05)		2007 (8.16)	1507 (8.73)		285 (0.98)	122 (0.73)	
Surgery only	11573 (20.61)	8273 (21.64)		6617 (26.90)	4780 (27.70)		13811 (47.56)	8220 (49.34)	
Cancer Stage (n, %)			<0.001			0.99			<0.001
In situ	8971 (15.97)	6389 (16.71)		638 (2.59)	407 (2.36)		1484 (5.11)	986 (5.92)	
Localized	33182 (59.09)	23090 (60.40)		10110 (41.07)	7537 (43.68)		13016 (44.83)	7864 (47.20)	
Regional	11367 (20.24)	7176 (18.77)		7412 (30.13)	5063 (29.34)		10908 (37.57)	5795 (34.78)	
Distant	1946 (3.47)	1083 (2.83)		5887 (23.93)	3776 (21.88)		2770 (9.54)	1389 (8.34)	
Comorbidity Score (n, %)			<0.001			0.98			<0.001
0	23633 (42.08)	10521 (21.52)		4914 (19.97)	2117 (12.27)		8892 (30.62)	2924 (17.55)	
1	15194 (27.06)	9469 (24.77)		6836 (27.79)	3567 (20.67)		7664 (26.39)	3490 (20.95)	
>=2	17330 (30.86)	18240 (47.71)		12852 (52.24)	11571 (67.06)		12481 (42.98)	10246 (61.50)	
Year of Cancer Diagnosis (n, %)			<0.001			0.99			<0.001
2007	4876 (8.68)	2790 (7.30)		2221 (9.03)	1257 (7.28)		3191 (10.99)	1503 (9.02)	
2008	4834 (8.61)	2892 (7.56)		2169 (8.82)	1308 (7.58)		3008 (10.36)	1492 (8.96)	
2009	4719 (8.40)	2908 (7.61)		2154 (8.76)	1313 (7.61)		2812 (9.68)	1429 (8.58)	
2010	4614 (8.22)	3002 (7.85)		2060 (8.37)	1351 (7.83)		2626 (9.04)	1506 (9.04)	
2011	4779 (8.51)	3154 (8.25)		2230 (9.06)	1446 (8.38)		2620 (9.02)	1507 (9.05)	
2012	5149 (9.17)	3471 (9.08)		2149 (8.74)	1558 (9.03)		2676 (9.22)	1545 (9.27)	
2013	5528 (9.84)	3932 (10.29)		2279 (9.26)	1782 (10.33)		2696 (9.28)	1668 (10.01)	
2014	5341 (9.51)	4155 (10.87)		2298 (9.34)	1854 (10.74)		2439 (8.40)	1579 (9.48)	
2015	5202 (9.26)	4433 (11.60)		2236 (9.34)	2008 (11.64)		2329 (8.02)	1718 (10.31)	
2016	6331 (11.27)	3904 (10.21)		2644 (10.75)	1757 (10.18)		2650 (9.13)	1442 (8.66)	
2017	4874 (8.52)	3589 (9.39)		2162 (8.79)	1621 (9.39)		1990 (6.85)	1271 (7.63)	
Chronic treatment-related pain (n, %)	5723 (10.19)	7101 (18.57)	<0.001	4409 (17.92)	4627 (26.82)	<0.001	3288 (11.32)	3243 (19.47)	<0.001
Neoplasm pain (n, %)	2535 (4.51)	2118 (5.54)	<0.001	3150 (12.80)	2494 (14.45)	<0.001	2080 (7.16)	1440 (8.64)	<0.001

Most patients were 66−70 years old, female, White, married, and were residents of a metropolitan area in both groups. The comorbidity scores were higher in patients with CNCP and cancer, i.e., 47.7% of CNCP in breast cancer cohort, 67.1% of CNCP in lung cancer cohort, and 61.5% of CNCP in colorectal cancer cohort had comorbidity scores of ≥2 compared to no-CNCP group. Chronic cancer-treatment-related pain was more common among patients with CNCP compared to patients without CNCP (breast cancer cohort = 18.5% vs 10.1%, p < 0.001, lung cancer cohort = 26.8% vs 17.9%, p < 0.001, colorectal cancer cohort = 19.4% vs 11.3%, p < 0.001). A higher proportion of patients in CNCP group had neoplasm related pain and compared to patients without CNCP in all cancer cohorts (breast cancer cohort = 5.5% vs 4.5%, p < 0.001, lung cancer cohort = 14.4% vs 12.8%, p < 0.001, colorectal cancer cohort = 8.6% vs 7.1%, p < 0.001). The diagnosis rate of chronic pain was consistent in all cancer cohorts throughout the years 2007–2017, ranging from 7.3% − 11.6% in breast cancer cohort, 7.2% − 11.6% in lung cancer cohort, and 7.6% − 10.3% in colorectal cancer cohort.

Study sample after IPTW had all covariates balanced between the CNCP and no-CNCP groups within all cancer cohorts (SMD < 10%) (S2 Table).

### Patterns of opioid prescriptions

S3 Table compares the patterns of opioid prescription among cancer patients within CNCP and no-CNCP before weighting. The overall rate of opioid use was 72.2% in the unweighted cohort. Among patients prescribed with opioids, 14.8% were prescribed ≥90 MME per day, 17.2% were prescribed 50- < 90 MME per day, and the remaining 67.9% were prescribed <50 MME per day.

The proportion of patients with any opioid use was higher among CNCP group (76.2%) compared to no-CNCP group (69.6%). Patients in the CNCP cohort were prescribed higher doses of opioids (≥90 MME/day) (12.2%) compared to those in the no-CNCP group (9.7%). Both the average number of opioid prescriptions and chronic opioid use were also higher among CNCP group compared to no-CNCP (S3 Table)

Similarly, any opioid use was more common among patients diagnosed with lung cancer (79.2%) followed by colorectal cancer (67.6%) and breast cancer (60.2%) in patients with CNCP. More patients with lung cancer (15.1%) were prescribed high doses of opioids compared to patients with breast cancer (11.4%) and colorectal cancer in the CNCP cohort (10.9%). The median number of opioid prescriptions filled was higher in patients with lung cancer (4.0) compared to patients with colorectal (3.0) and breast cancer (2.0) within CNCP cohort. Similarly, patients with lung cancer (30.8%) had higher chronic opioid use compared to patients with colorectal (20.9%) and breast cancer (18.3%) in the CNCP cohort.

### Results of regressions After IPTW adjustment

Table 2 shows the result of logistic and poisson regressions after IPTW adjustment. Similarly, patients with lung cancer and CNCP had higher odds of any opioid use compared to patients without CNCP (OR 1.55;95% CI 1.49–1.63; p < 0.001), and patients with breast cancer and CNCP had higher odds of chronic opioid use (OR 2.82; 95% CI 2.70–2.94; p < 0.001). Patients with breast, lung, or colorectal cancer with CNCP had lower odds of high dose opioid prescriptions compared to patients without CNCP (OR 0.82; 95% CI 0.8–0.84; p < 0.001, OR 0.68; 95% CI 0.66–0.71; p < 0.001, OR0.74; 95% CI 0.71–0.77; p < 0.001, respectively) Similarly, patients with breast, lung, and colorectal cancer with CNCP had 46.32%, 44.24%, 43.15% higher number of opioid prescriptions filled annually (estimate 0.46; 95% CI 0.45–0.46; p < 0.001, estimate 0.43; 95% CI 0.42–0.44; p < 0.001).

**Table 2:** Results of regression after weighting.

Patterns of opioids prescriptions	Odds ratio (95% CI	p-value
**Opioid Use**		
Patients with CNCP and breast cancer*	1.27 (1.23-1.31)	<0.001
Patients with CNCP and lung cancer^β^	1.55 (1.49-1.63)	<0.001
Patients with CNCP and colorectal cancer^µ^	1.41(1.35-1.47)	<0.001
**Higher dose of opioid prescriptions (Average MME per day >= 90)**		
Patients with CNCP and breast cancer*	0.82 (0.80-0.84)	<0.001
Patients with CNCP and lung cancer^β^	0.68 (0.66-0.71)	<0.001
Patients with CNCP and colorectal cancer^µ^	0.74 (0.71-0.77)	<0.001
**Chronic opioid use**		
Patients with CNCP and breast cancer*	2.77 (2.65-2.89)	<0.001
Patients with CNCP and lung cancer^β^	2.39 (2.27-2.51)	<0.001
Patients with CNCP and colorectal cancer^µ^	2.39 (2.25-2.53)	<0.001
**Number of opioid prescriptions filled**	**Estimate (95% CI)**	**p-value**
Patients with CNCP and breast cancer*	0.46 (0.45-0.47)	<0.001
Patients with CNCP and lung cancer^β^	0.44 (0.43–0.45)	<0.001
Patients with CNCP and colorectal cancer^µ^	0.43 (0.42-0.45)	<0.001

CNCP = Chronic non-cancer pain, MME = Morphine milligram equivalent. * represents the reference group was patients with breast cancer without CNCP, ^β^ represents the reference group was patients with lung cancer without CNCP, and ^µ^represents the reference group was patients with colorectal cancer without CNCP.

## Discussion

One of the key challenges associated with opioid prescriptions in elderly cancer patients is the risk of adverse events and polypharmacy [57,77]. While cancer treatment modalities continue to improve, life expectancy has increased for cancer survivors. Additionally, elderly patients with cancer and comorbid CNCP may have higher opioid prescriptions. Therefore, it is important to consider opioid prescribing patterns as they are associated with potential negative outcomes. In this retrospective cohort study, we aimed to evaluate the impact of comorbid CNCP on opioid prescriptions patterns in terms of any opioid use, high-dose opioid use, total count of opioid prescriptions filled, and chronic opioid use, among patients with breast, lung and colorectal cancer.

To begin, patients with CNCP and cancer demonstrated higher odds of receiving any opioid prescriptions, chronic opioid use, as well as greater number of opioid prescriptions filled, which may increase their risk of opioid misuse, opioid overdose, and addiction. Previous studies have explored the CNCP and opioid use among cancer survivors excluding active cancer patients [43,78–80]. Within studies including active cancer patients, the focus has mostly been on breakthrough pain, cancer pain, or cancer-treatment-related pain [63,64,81,82]. The study conducted by Beck *et al.* (2024) among patients with cancer presenting to emergency department with acute pain showed that pre-existing CNCP was associated with greater use of opioids in the hospital which was consistent with the findings of our study [83]. Further, the study by Salz *et al.* (2019) described the trend of opioid use among elderly breast, lung, and colorectal cancer survivors post-diagnosis from one to six years following cancer diagnosis [43]. However, cancer patients were compared to non-cancer patients and impact of CNCP were not explored. Our study fills the critical gap in understanding how CNCP influences opioid prescribing in this population, which is essential for optimizing pain management while minimizing inappropriate use. Furthermore, other non-pharmacological approaches need to be explored and considered for the treatment of CNCP.

Confounding by indication is a potential concern for the high-dose opioid use, as patients with comorbid CNCP may have greater baseline pain and opioid tolerance that could result in prescription of higher dose of opioids. However, results of our study showed lower odds of high-dose opioids but higher odds of chronic opioid use among cancer patients with CNCP. The lower odds of high-dose opioid prescriptions in patients with CNCP suggest that clinicians may adopt a more cautious approach to dose escalation and are aware of the risks of opioid misuse in individuals with pre-existing CNCP [46]. It may also suggest better titration or monitoring practices in this population, or the use of multimodal pain management strategies aimed at minimizing opioid dose escalation [26]. Despite the awareness and multiple approach, chronic opioid use among this population remains unavoidable. Future research should explore the trajectories of opioid use among patients with comorbid CNCP during active cancer treatment.

The findings of the study highlight the burden of comorbidity of chronic pain in the management of pain in cancer patients. While the major focus in other studies and guidelines is mostly on neoplastic pain and cancer-treatment-related pain, [26,38,64,81,82,84] non-cancer chronic pain also adds complications to patient health status and treatment. Further, the guidelines for cancer pain have limited recommendations for CNCP and focused on cancer and cancer-treatment-related pain only [26,42]. Considering the potential for opioid misuse, our study illustrates the importance of accounting for such complexities in the guidelines based on safety and effectiveness evidence to improve the optimization of pain management and treatment among patients with CNCP and cancer. Therefore, integrated guidelines for CNCP and cancer pain management are required to address the complexities of pain management in patients with both conditions.

After adjusting clinical variables such as cancer stage, neoplasm pain, and chronic cancer-treatment-related pain, our study indicates that patients with lung cancer and CNCP have significantly higher odds of any opioid prescription. Additionally, patients with breast cancer and CNCP exhibit chronic opioid use and receive a higher number of opioid prescriptions. The differences may be due to factors such as disease trajectories and pain characteristics. Lung cancer is frequently diagnosed with advanced stage and is often associated with acute, severe, and rapidly progressing pain, which may prompt the increased any opioid use [85–87]. However, breast cancer is generally associated with longer survival and higher likelihood of survivorship [88]. Patients with breast cancer may experience pain during the survivorship period, which may increase prolonged opioid use. Further, pain perceptions, patient-clinician communication, and provider prescribing behavior can be other factors causing the difference in opioid prescriptions among patients with lung and breast cancer [89,90]. The result underlines the need for extra vigilance for the patients with lung and breast cancer when managing opioid therapy. Adequate pain treatment needs to be ensured while minimizing the risk of opioid-related adverse events and avoiding both undertreatment and overprescribing. Future studies should further examine other opioid-related outcomes in patients with lung and breast cancer and CNCP. Further, the use of urine drug tests and pharmacogenomics before prescribing opioids can help stratify patients based on their likelihood of negative outcomes and could personalize treatment that considers individual risk factors, metabolic profiles, genetic profile, and potential to misuse, thereby guiding more appropriate opioid selection, dosing, and monitoring strategies to their individual needs.

There are a few limitations to our study. First, while we have discussed pain based on the source of pain, classification based on etiology of pain, such as nociceptive pain and neuropathic pain, were not considered. Second, we used claims data for prescribing, for which we assumed that patients were taking these medications whenever there were claims for opioids. However, actual patient adherence could not be verified, and it is possible that some patients did not take the medications as prescribed. Third, we used SEER registry data for the treatment modality variables. While the SEER registry database provides robust and substantial clinical and demographic variables, the availability and completeness of chemotherapy and radiotherapy information are limited. This may lead to misclassification or underestimation of treatment exposure [91]. Fourth, we included patients with incident cancer, we did not exclude if patients had recurrent cancer later during the study period. Even though we focused on the impact of comorbid CNCP in opioid prescribing, the presence of recurrent cancer may influence pain trajectories and opioid use. Fifth, we did not specifically exclude opioid formulations prescribed for non-pain reasons such as cough suppressants. These prescriptions are substantially lower doses and are prescribed for shorter durations and their inclusion may have modestly influenced estimates of any opioid use and number of prescription fills. Next, opioid use was identified using Medicare part-D claims and a predefined list of commonly prescribed opioids. Therefore, opioid administered under Medicare Part B and opioid formulations not included in the study definition may not have been captured, potentially leading to underestimation of overall opioid exposure. Last, although we based our definition of CNCP on a validated algorithm, we cannot rule out the chances of potential misclassification. ICD-11 provides a standardized framework to differentiate pain due to tumor, pain due to cancer treatment, and comorbid pain conditions [92]. Future studies should incorporate ICD-11 classification of pain to minimize the potential misclassification of different pain conditions. However, there are strengths of the study. one strength lies in the study’s novelty. Previous studies mostly focused on cancer and cancer-treatment-related pain in cancer patients. This is the first study to explore opioid prescribing in patients with dual diagnosis (i.e., CNCP and active cancer together). Results of the study provides insight for healthcare stakeholders to understand the impact of CNCP as a comorbidity in active cancer patients in designing the pain management strategies and interventions. Additionally, the study used SEER-Medicare data with large sample size which increases the generalizability of the study.

## Conclusion

In conclusion, pre-existing CNCP in patients with cancer is associated with chronic opioid use. Opioid prescriptions should be carefully evaluated to minimize the risk of opioid-related harms of opioid prescriptions in such populations. Future study should focus on personalized pain management strategies and effectiveness of non-pharmacological approaches in such populations.

## Supporting information

S1 Table ICD codes and generic names. (S1_File.PDF)

S2 Table Standardized differences in percent before and after IPTW. (S2_File.PDF)

S3 Table Patterns of opioid prescriptions before weighting. (S3_File.PDF)
