# Supplementary material for: Pattern of opioid prescriptions among patients with breast, lung, and colorectal cancer diagnosed with pre-existing chronic non-cancer pain
Source: PLoS One. 2026 Jul 29;21(7):e0352907. doi: 10.1371/journal.pone.0352907 (PMC13419219; doi:10.1371/journal.pone.0352907)
Supplement: S1 Table — (S1_File.PDF) [file pone.0352907.s001.pdf]

S1 Table: ICD Codes and Generic Names

	ICD-9-CM	ICD-10-CM
Neoplasm Pain	338.3	G89.3
**Chronic non-cancer pain**		
Chronic Pain	338.2	G89.2
Chronic Pain due to Trauma	338.21	G89.21
Other Chronic Pain	338.29	G89.29
Chronic pain syndrome	338.4	G89.4
Psychogenic Pain Nos	307.8	F45.41
Psychogenic Pain Nec	307.89	F45.42
Central Pain Syndrome	338	G89.0
Joint Pain, Shoulder	719.41	M25.519
		M25.511
		M25.512
Joint Pain Multiple Sites	719.49	
Joint Pain, Hip or Thigh	719.45	M25.559
		M25.551
		M25.552
Pain Joint- Lower Leg, Knee	719.46	M25.569
Joint Pain- Ankle	719.47	M25.571
		M25.572
		M25.579
Anklosing Spondylitis	720	M45.3
Inflammation of Sacroiliac Joint NOS	720.2	M45.4
		M45.5
		M45.6
		M45.7

		M45.2
		M45
		M45.9
		M45.8
Inflam Spondylopathy Nos	720.9	M46.95
		M46.97
		M46.90
		M46.91
		M46.93
		M46.94
		M46.99
		M46.92
		M46.98
Anterior Spinal Artery Compression Syndrome	721	M47.812
		M47.811
		M47.813
Thoracic Arthritis	721.1	M47.016
		M47.019
		M47.012
		M47.013
		M47.014
		M47.015
		M47.011
		M47.022
		M47.021
		M47.12
		M47.11
		M47.13
		M47.814
Lumbar and Sacral Arthritis	721.2	M47.815
Spond Compr Thor Sp Cord	721.3	M47.817
		M47.818
		M47.816
Lumbar Spondylosis with Myelopathy	721.41	M47.14
		M47.15

Ankyl Vert Hyperostosis	721.42	M47.16
Other Allied Disorders of Spine	721.6	M48.10
Degenerative Spinal Arthritis	721.8	M48.9
Spondylogenic Compression of Spinal Cord NOS	721.9	M47.9
		M47.819
Cervical Disc Displacement	721.91	M47.90
		M47.892
		M47.893
		M47.894
		M47.895
		M47.896
		M47.897
		M47.899
		M47.10
		M47.12
		M47.13
		M47.14
		M47.15
		M47.16
		M47.20
		M47.22
		M47.23
		M47.24
		M47.25
		M47.26
		M47.27
		M47.898
		M47.11
		M47.28
		M47.812
		M47.813
		M47.814
		M47.815
		M47.816

		M47.817
		M47.818
		M47.819
		M47.891
		M47.811
	722	M50.20
		M50.21
		M50.221
Myelopathy	722.1	M51.24
Myelopathy	722.11	M51.25
		M51.9
Disc Displacement Nos	722.2	
Schmorl's Disease	722.3	M51.44
Schmorl's Nodes of Thoracic region	722.31	M51.45
		M51.46
Schmorl's Nodes of Lumbar Region	722.32	M51.47
Schmorl's Nodes of Other Region NEC	722.39	M50.30
Cervical Disc Degen	722.4	M51.34
Degeneration of Thoracic Intervertebral Disc	722.51	M51.35
Degeneration of Intervertebral Disc NOS	722.6	
Disc Dis W Myelopath Nos	722.7	M50.00
Cervical Disc Dis W Myelopath	722.71	M51.06
Myelopathy	722.73	
Post laminectomy Synd Nos	722.8	M96.1
Postlaminect Synd-Cerv	722.81	

Postlaminect Synd-Thorac	722.82	
Postlaminect Synd-Lumbar	722.83	
Calcification of Intervertebral Cartilage Or Disc NOS	722.9	M50.80
Region Calcification of Intervertebral Cartilage	722.91	M50.90
		M46.45
		M51.9
Thoracic Region	722.92	M51.84
		M51.85
Region	722.93	
Cervical Spinal Stenosis	723	M50.021
Cervicalgia	723.1	M46.47
		M54.2
Cervicobrachial Syndrome	723.3	M53.1
Brachial Neuritis NOS	723.4	M54.12
		M54.13
Contracture of Neck NOS	723.5	M43.6
Panniculitis Affecting Neck	723.6	M54.02
Ossification Cerv Lig	723.7	
Cervical Syndrome NEC	723.8	M53.82
Disorder of Cervical Region NEC	723.9	
Spinal Stenosis Nos	724	

Spinal Stenosis of Thoracic Region	724.01	M54.6
Spinal Stenosis of Lumbar Region	724.02	M48.061
Spinal Stenosis NOS	724.09	M48.08
Pain in Thoracic Spine	724.1	M54.6
Low Back Pain	724.2	M54.5
Cotungo’s Disease	724.3	M54.30
Lumbosacral Neuritis NOS	724.4	M54.14
		M54.15
		M54.16
		M54.17
Back Pain	724.5	M54.89
		M54.9
Ankylosis of Lumbosacral Joint	724.6	M43.27
		M43.28
		M53.2X7
		M53.3
Disorder of Coccyx NOS	724.7	
Coccydynia	724.79	
Ossification of Posterior Longitudinal Ligament NOS	724.8	M54.08
Ankylosis of Spine NOS	724.9	M43.8X9
		M53.9
Fibrositis NOS	729	
Fibromyalgia	729.1	M60.9
		M79.7
Neuralgia NOS	729.2	M54.10
		M79.2
Fasciitis	729.4	M72.9
Pain in Limb	729.5	M79.609
		M79.606
		M79.601
		M79.602

		M79.605
		M79.604
		M79.676
		M79.659
		M79.669
		M79.646
		M79.651
		M79.652
		M79.661
		M79.662
		M79.606
**Cancer treatment related pain**		
**Surgery**		
Intercostal Neuralgia	353.8	G54.8
Lymphedema	457.1	I97.2
	457	I89.0
Neuroma pain	355.6	G57.60
Pain related to breast implants/reconstruction	996.54	T85.49XA
Phantom pain	353.6	G54.6
Postmastectomy pain	338.28	G89.28
Post-thoracotomy pain	338.22	G89.22
**Radiation**		
Chest pain/tightness	786.59	R07.82
		R07.89
	780.96	R52
	786.5	R07.9
	786.51	R07.2
	786.52	R07.81
Cystities	595.82	N30.40
		N30.41
Enteritis/proctitis	556.2	K51.20
Fibrosis of skin or myofascia	709.8	L94.2
		L98.8
Fistula formation	996.1	T82.590A
	442.9	I72.9
Myelopathy	336.8	G98.89
Osteoradionecrosis	526.89	M27.2
		M27.8

Peripheral nerve entrapment	337	G90.09
	337.09	
Plexopathies	353.8	G54.8
	728.9	M62.9
GI, abdominal, other adhesions in the radiation field	560.81	K56.50
		K56.51
		K56.52
**Hormonal therapy**		
Arthralgia/myalgia	729.1	
Muscle cramps/spasms	728.85	M62.40
		M62.838
Carpal tunnel syndrome	354	G56.00
Trigger finger	727.03	M65.30
**Chemotherapy**		
Arthralgia/myalgia	729.1	
	729.2	
Osteoporosis	733.09	M81.8
Osteonecrosis		M87.1
Chemotherapy-indiced peripheral neuropathy	357.6	G62.0
Muscle cramps	728.85	M62.40
		M62.838
**Opioids**	**Generic names:** Codeine, Fentanyl, Hydrocodone,Hydromorphone, Methadone, Morphine, Oxycodone, Oxymorphone, Tramadol, Tapentadol, Meperidine, Butorphanol, Levorphanol, and Pentazocine	
