# Supplementary material for: Pattern of opioid prescriptions among patients with breast, lung, and colorectal cancer diagnosed with pre-existing chronic non-cancer pain
Source: PLoS One. 2026 Jul 29;21(7):e0352907. doi: 10.1371/journal.pone.0352907 (PMC13419219; doi:10.1371/journal.pone.0352907)
Supplement: S2 Table — (S2_File.PDF) [file pone.0352907.s002.pdf]

S2 Table: Standardized Differences in Percent Before and After IPTW

Sociodemographic characteritistics	Breast Cancer		Lung Cancer		Colorectal Cancer	
	Before IPTW	After IPTW	Before IPTW	After IPTW	Before IPTW	After IPTW
	Standardized difference in Percent (%)	Standardized difference in Percent(%)	Standardized difference in Percent (%)	Standardized difference in Percent (%)	Standardized difference in Percent (%)	Standardized difference in Percent (%)
**Age**						
66-70 age	-8.50	0.02	-4.99	0.06	-9.15	-0.18
71-74 age	-2.59	-0.01	-4.98	-0.03	-5.08	0.19
75-78 age	1.93	0.03	1.31	-0.01	-0.59	-0.04
79-82 age	3.70	-0.02	3.93	-0.06	4.20	-0.04
83 and above age	7.91	-0.03	7.76	0.03	10.12	0.07
**Sex***	-2.74	-0.01	15.6	-0.15	-14.78	0.13
**Race**						
White	2.10	0.02	2.43	0.11	4.74	0.24
Black	1.85	-0.06	0.67	-0.13	-0.86	-0.07
Others	-6.17	0.05	-4.58	-0.01	-5.44	-0.33
**Ethnicity***	-0.13	-0.09	-0.07	-0.11	-2.98	-0.01
**Martial Status**						
Never married	0.15	0.67	-0.97	-0.40	-2.17	-0.71
Married	-5.66	-0.04	-7.04	0.06	-8.41	1.69
Wid/Sep/Div	3.26	0.04	5.17	0.85	4.28	-0.13
**Metropolitian status**						
Metro	3.55	-0.30	2.29	0.02	4.57	-0.47
Non-metro	-3.34	0.31	-2.63	-0.03	-4.36	0.44
Rural	-1.06	0.03	0.62	0.00	-1.19	0.14
**Treatment Modality**						
No treatment	1.66	0.17	1.86	-0.01	0.99	-0.15
Chemotherapy only	0.01	0.03	-2.40	0.17	-1.98	0.32

Surgery only	2.53	-0.01	1.81	0.04	3.55	0.27
Radiotherapy only	-1.13	-0.01	2.07	0.01	-2.70	0.07
More than one treatment	-4.63	0.06	-7.21	-0.14	-11.48	0.11
**Cancer Stage**						
In situ	1.99	0.01	-1.51	-0.07	3.54	-0.37
Localized	2.67	0.08	5.23	-0.08	4.77	-0.06
Regional	-3.71	-0.06	-1.72	0.10	-5.79	0.14
Distant	-3.62	-0.06	-4.87	0.00	-4.21	0.28
**Charlson comorbidity score**						
0	-30.94	-0.07	-21.07	-0.05	-30.93	0.06
1	-5.22	0.03	-16.66	0.02	-12.84	-0.10
>=2	35.03	0.05	30.56	0.02	37.73	0.04
**Year of Diagnois**						
2007	-5.11	-0.09	-6.37	0.13	-6.56	-0.19
2008	-3.83	-0.03	-4.51	-0.07	-4.75	-0.46
2009	-2.94	-0.13	-4.18	-0.08	-3.84	-0.38
2010	-1.34	-0.05	-1.99	0.12	-0.01	-0.02
2011	-0.94	-0.19	-2.42	-0.21	0.08	0.16
2012	-0.31	0.00	1.03	-0.07	0.20	0.13
2013	1.47	-0.02	3.58	-0.04	2.46	0.08
2014	4.49	0.02	4.67	-0.01	3.78	-0.14
2015	7.64	-0.04	8.37	-0.08	7.95	0.11
2016	-3.43	0.21	-1.84	0.16	-1.65	0.36
2017	3.04	0.28	2.11	0.12	2.99	0.42
**Chronic Treatment Pain***	24.06	-0.01	21.47	-0.05	22.71	-0.14
**Neoplasm Pain***	4.70	-0.04	4.81	-0.01	5.49	-0.03

For binary variables, only one Standard difference was calculated for the variable and for the nominal variables, standard difference was calculated for each group. * represents binary variables. IPTW- Inverse probability of treatment weighting.
