# Supplementary material for: Pattern of opioid prescriptions among patients with breast, lung, and colorectal cancer diagnosed with pre-existing chronic non-cancer pain
Source: PLoS One. 2026 Jul 29;21(7):e0352907. doi: 10.1371/journal.pone.0352907 (PMC13419219; doi:10.1371/journal.pone.0352907)
Supplement: S3 Table — (S3_File.PDF) [file pone.0352907.s003.pdf]

S3 Table: Patterns of Opioid Prescriptions Before Weighting

	Patients without CNCP (N=109796)				Patients with CNCP (N=72145)				p-value
	Overall	Breast Cancer (N=56157)	Lung Cancer (N=24602)	Colorectal cancer (N=29037)	Overall	Breast Cancer (N=38230)	Lung Cancer (N=17255)	Colorectal Cancer (N=16660)	
Opioid Use*Ł (n, %)	76417 (69.60)	41517 (73.93)	17321 (70.40)	17579 (60.54)	54960 (76.18)	23015 (60.20)	13660 (79.17)	11264 (67.61)	<0.001
Average MME per day*Ł (n, %)									<0.001
<50 MME/day	55979 (50.36)	31061 (55.31)	11925 (48.47)	12993 (44.75)	41105 (56.67)	23024 (60.20)	9456 (54.80)	8643 (51.82)	
50-<90 MME/day	18822 (17.00)	9816 (17.48)	4803 (19.52)	4203 (14.47)	12332 (16.85)	6428 (16.81)	3561 (20.64)	2342 (14.06)	
≥90 MME/day	1616 (2.24)	640 (1.14)	593 (2.41)	383 (1.32)	1523 (2.62)	593 (1.55)	643 (3.73)	287 (1.72)	
No. of opioid prescriptions*Ł (Median, IQR)	1.00 (1.00-3.00)	1.00 (1.00-2.00)	2.00 (1.00-5.00)	1.00 (1.00-3.00)	3.00 (1.00-7.00)	2.00 (1.00-6.00)	4.00 (2.00-11.00)	3.00 (1.00-7.00)	<0.001
Chronic opioid use *Ł (n, %)	9332 (8.67)	3556 (6.33)	3412 (13.87)	2550 (8.78)	15654 (21.92)	7002 (18.32)	5321 (30.84)	3490 (20.95)	<0.001

CNCP = Chronic non-cancer pain, MME = Morphine milligram equivalent
p-value was calculated between CNCP and no-CNCP group. * Represents p-value <0.001 between cancer types among CNCP cohort and Ł represents p-value <0.001 between cancer types among no-CNCP cohort.
